# RP11-Derived Long Non-Coding RNAs in Hepatocellular Carcinoma: Hidden Treasures in Plain Sight

**DOI:** 10.32604/or.2025.072240

**Published:** 2025-12-30

**Authors:** Se Ha Jang, Hyung Seok Kim, Jung Woo Eun

**Affiliations:** 1 Department of Gastroenterology, Ajou University School of Medicine, 164 Worldcup-ro, Yeongtong-gu, Suwon, 16499, Republic of Korea; 2 Department of Biomedical Sciences, Ajou University Graduate School of Medicine, 164 Worldcup-ro, Yeongtong-gu, Suwon, 16499, Republic of Korea; 3 Department of Biochemistry, Kosin University College of Medicine, Seo-gu, Busan, 49267, Republic of Korea

**Keywords:** Hepatocellular carcinoma, long non-coding RNA, RP11-derived lncRNA, biomarker, therapeutic target

## Abstract

Hepatocellular carcinoma (HCC) remains one of the most prevalent and lethal malignancies worldwide. Long non-coding RNAs (lncRNAs) have emerged as crucial regulators of gene expression and cancer progression, yet the functional diversity of RP11-derived lncRNAs—originally mapped to bacterial artificial chromosome (BAC) clones from the Roswell Park Cancer Institute—has only recently begun to be appreciated. This mini-review aims to systematically synthesize current findings on RP11-derived lncRNAs in HCC, outlining their genomic origins, molecular mechanisms, and biological significance. We highlight their roles in metabolic reprogramming, microRNA network modulation, and tumor progression, as well as their diagnostic and prognostic value in tissue and serum-based analyses. Finally, we discuss therapeutic opportunities and propose future directions to translate RP11-derived lncRNAs into clinically actionable biomarkers and targets for precision liver cancer therapy.

## 1 Introduction

Long non-coding RNAs (lncRNAs)—transcripts longer than 200 nucleotides with no protein-coding potential—are key regulators of gene expression. They function through chromatin remodeling, transcriptional and post-transcriptional regulation, and interactions with proteins and other RNAs [1]. A particularly large family of lncRNAs carries the “RP” prefix, which denotes transcripts originally mapped to bacterial artificial chromosome (BAC) clones from the Roswell Park Cancer Institute (RPCI) libraries during the Human Genome Project [2,3]. In our compendium of total gene entries, 10,430 bear RP designations (≈17%); among these, the RP11 subset is the largest single group. RP11-derived lncRNAs are heterogeneous in origin and function, with members reported to act as oncogenes or tumor suppressors depending on cellular context [4–6].

While numerous RP11-derived lncRNAs are implicated in hepatocellular carcinoma (HCC), prior reviews have either addressed lncRNAs broadly or focused on other functional subclasses. Accordingly, this review provides the first critical synthesis dedicated specifically to the RP11 family in HCC. Moving beyond a simple catalog, our primary goal is to identify and analyze the convergent molecular mechanisms that unify the roles of these diverse transcripts. By evaluating the evidence from this focused perspective, we aim to establish a conceptual framework for prioritizing the most clinically and functionally relevant RP11 lncRNAs as biomarkers and therapeutic targets.

## 2 RP11 lncRNAs: Origins and Classification

The “RP” naming convention originated during the clone-based phase of the Human Genome Project, when the Roswell Park Cancer Institute constructed human bacterial artificial chromosome libraries to assemble the physical map for sequencing [2]. In this system, “RP” stands for Roswell Park, and the subsequent numeral (e.g., RP1, RP4, RP5, RP11) identifies a specific library prepared from a defined donor and protocol. This designation does not imply gene function, biotype, or genomic position. BAC libraries were generated from high-molecular-weight genomic DNA (~100–200 kb inserts) ligated into BAC vectors, transformed into *Escherichia coli*, and arrayed in microtiter plates; each clone was assigned a plate–row–column address that persists as the clone barcode (e.g., “34P13”) [2,7]. For example, RP-style symbols follow the pattern RP11-34P13.3, where “RP11” denotes the BAC library (RPCI-11), “34P13” is the plate–row–column barcode of the clone (plate 34, row P, column 13), and the terminal “.3” is the Havana/GENCODE gene-model index within that clone interval (not an isoform label). Additional qualifiers such as -AS1 (antisense), -IT1 (intronic transcript), -OT1 (overlapping transcript), and -P1 (pseudogene) describe genomic context rather than origin [2,8,9].

**Figure 1:**
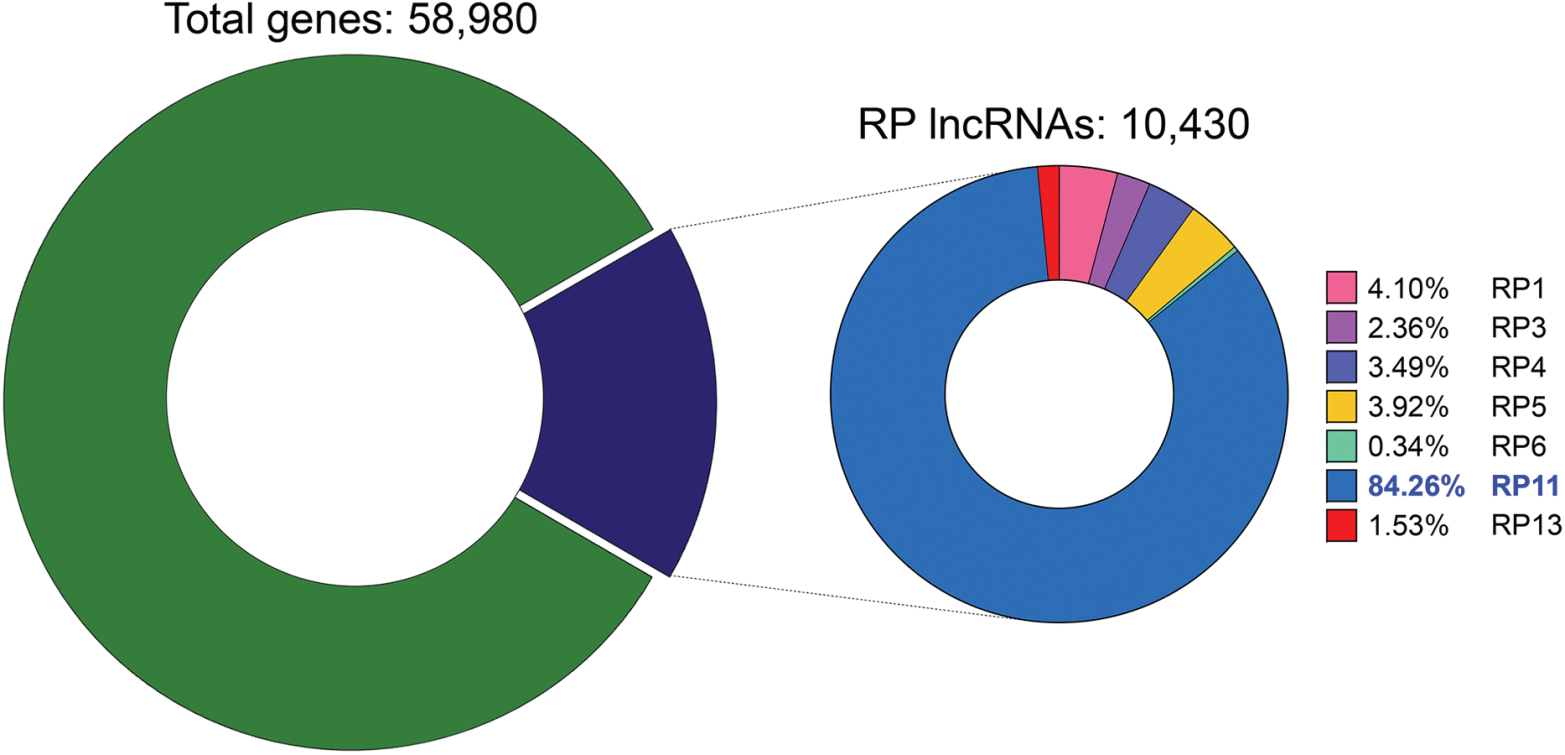
Distribution of RP-designated lncRNAs within the human gene compendium. The left donut chart shows that RP-designated long non-coding RNAs (lncRNAs, purple) constitute 17.7% (10,430) of all 58,980 annotated human genes (green). The right donut chart details the distribution of these RP lncRNAs based on their originating Roswell Park Cancer Institute (RPCI) bacterial artificial chromosome (BAC) library. The RP11 library is the largest contributor, accounting for 84.26% (8788 transcripts), followed by RP1 with 4.10% (428), RP5 with 3.92% (409), RP4 with 3.49% (364), RP3 with 2.36% (246), RP13 with 1.53% (160), and RP6 with 0.34% (35)

In our compendium of total gene entries, 10,430 carry an RP designation, underscoring how extensively RP clones shaped human genome annotation. The RP11 subset alone contributes 8788 of these entries (≈84.3%), making it the predominant group (Fig. 1).

Consequently, research attention has increasingly focused on RP11-named transcripts. Nevertheless, many of these remain poorly characterized beyond their genomic location. Consistent with their clone-anchored origins, the majority are long non-coding RNAs lacking protein-coding potential. These entities are now recognized as central regulators in liver cancer biology, yet they still require systematic functional and clinical validation.

## 3 RP11-Designated lncRNAs across Cancers

lncRNAs are widely implicated in oncogenesis and tumor progression across diverse cancer types. Within this landscape, transcripts with the “RP11” prefix—a legacy of clone-based human genome annotation—have emerged as recurrently dysregulated molecules with context-dependent functions [10,11]. Although the RP11 label itself does not imply a specific biology, multiple RP11-designated loci show reproducible associations with cancer phenotypes and patient outcomes, prompting increasing interest in their functional roles. Recent studies have identified the dysregulation of RP11 lncRNAs in various cancers, revealing their potential involvement in tumor initiation, progression, and therapeutic resistance (Table 1).

**Table 1:** Differential expression of RP11-lncRNAs in various cancers

Gene IDs	Cancer types	Expression	References
RP11-431M3.1	Colon cancer	Up	Bose et al., 2024 [12]
RP11-350G8.5	Multiple myeloma	Up	Grillone et al., 2024 [13]
RP11-417E7.1	Colon cancer	Up	Liu et al., 2024 [14]
RP11-874J12.4	Lung cancer	Up	Huang et al., 2024 [15]
RP11-295G20.2	Lung cancer	Up	Yu et al., 2024 [16]
RP11-363E7.4	Bladder cancer	Down	Silva et al., 2024 [17]
RP11-197K6.1	Colon cancer	Up	Wang et al., 2023 [18]
RP11-278A23.1	Colon cancer	Up	Kamikokura et al., 2024 [19]
RP11-89	Bladder cancer	Up	Luo et al., 2021 [20]
RP11-551L14.4	Breast cancer	Down	Wang et al., 2022 [21]
RP11-138J23.1	Gastric cancer	Up	Xu et al., 2022 [22]
RP11-283G6.5	Breast cancer	Down	Pei et al., 2021 [23]
RP11-1023L17.1	Prostate cancer	Up	Huang et al., 2022 [24]
RP11-5809.2	Lung cancer	Up	Miao et al., 2023 [25]

In colorectal cancer, RP11-431M3.1 is upregulated and promotes tumor progression by modulating the hypoxia-inducible factor 1 alpha (HIF1A)/miR-138 axis and Wnt/β-catenin signaling, thereby linking hypoxic responses to canonical proliferation/invasion programs [12]. In bladder cancer, RP11-89 functions as a competing endogenous RNA (ceRNA) that sponges miR-129-5p, thereby fostering ferroptosis resistance and facilitating tumorigenesis [20].

Conversely, tumor-suppressive RP11 transcripts have also been described. For example, RP11-551L14.4 is downregulated in breast cancer and restrains tumor progression by repressing miR-4472 [21]. In urothelial carcinoma, reduced RP11-363E7.4 expression correlates with an adverse prognosis, supporting its potential utility as a biomarker for risk stratification [17]. Therapy resistance and metastasis are additional recurring themes: in lung cancer, RP11-874J12.4 enhances erlotinib resistance via AXL upregulation [15], while RP11-295G20.2 expression in lung adenocarcinoma is associated with prognosis, suggesting a role in disease aggressiveness [16].

Mechanistically, RP11 lncRNAs operate through common functional modes, including: ceRNA/microRNA (miRNA) sponging; transcriptional co-regulation at promoters/enhancers; chromatin remodeling via the recruitment of modifying complexes; and post-transcriptional control through RNA binding protein–messenger RNA (mRNA) interactomes; and coupling to canonical oncogenic pathways [26]. Their functional polarity (as an oncogene or tumor suppressor) typically reflects their subcellular localization (e.g., cytoplasmic ceRNA vs. nuclear chromatin regulator), the specific target network engaged, and tissue-restricted transcription factor and RNA-binding protein availability [27]. Collectively, these observations indicate that RP11 lncRNAs participate in diverse, pathway-specific circuits that govern proliferation, invasiveness, cell-death vulnerability, drug response, and clinical trajectory across tumor types.

## 4 RP11 lncRNAs as Biomarkers in HCC

Dozens of RP11 lncRNAs have been proposed as biomarker candidates in HCC, but the clinical evidence supporting them varies substantially. A critical evaluation of the literature reveals a clear hierarchy: a small subset of candidates stands out, supported by studies in large clinical cohorts and detectable in minimally invasive samples like serum, positioning them as the most promising for clinical translation. A second tier of lncRNAs shows compelling mechanistic links but has so far only been validated in smaller patient sets or tissue samples alone. This section will synthesize the evidence for these candidates, focusing on those that have been demonstrated in clinical settings and show potential as viable biomarkers (Table 2).

**Table 2:** Differential expression of RP11-lncRNAs as Biomarkers in HCC

Gene IDs	Expression	Type	Platform	Cohort (n)	References
RP11-40C6.2	Up	Tissue, serum	qRT-PCR, RNA sequencing	193	[28]
RP11-286H15.1	Down	Tissue	qRT-PCR, FISH, RNA sequencing	451	[29]
RP11-466I1.1	Up	Tissue	qRT-PCR	83	[30]
		Serum			
RP11-156P1.3	Up	Tissue	qRT-PCR	123	[31]
		Serum			
RP11-284P20.2	Up	Tissue	qRT-PCR	27	[32]
RP11-439C15.4	Down	Tissue	qRT-PCR, RNA sequencing	479	[33]
RP11-465B22.3	Up	Tissue	RNA sequencing, qRT-PCR	470	[34]
RP11-556E13.1	Up	Tissue	Microarray, qRT-PCR	112	[35]
RP11-324I22.4	Up	Tissue	RNA sequencing	370	[36]
RP11-298O21.2	Up	Tissue	RNA sequencing, qRT-PCR	339	[37]
RP11-383H13.1	Up				
RP11-440G9.1	Up				
RP11-498C9.15	Up	Tissue	RNA sequencing	368	[38]
RP11-96A15.1	Up	Tissue	RNA sequencing, qRT-PCR	410	[39]
RP11-305F18.1	Up				
RP11-342M1.3	Up				
RP11-432J24.3	Up				
RP11-598F7.3	Up	Tissue	qRT-PCR	384	[40]
RP11-620J15.3	Up	Tissue	RNA sequencing	368	[41]
RP11-290F5.1	Up				
RP11-147L13.13	Up				
RP11-923I11.6	Up				
RP11-495K9.6	Up	Tissue	RNA sequencing	180	[42]
RP11-96O20.2	Up				
RP11-359K18.3	Up				
RP11-486O12.2	Up	Tissue	RNA sequencing	361	[43]
RP11-863K10.7	Down				
RP11-273G15.2	Down				
RP11-325L7.2	Down	Tissue	RNA sequencing	167	[44]
RP11-100L22.4	Down				
RP11-104L21.3	Up				
RP11-598D14.1	Down	Tissue	qRT-PCR	50	[45]
RP11-363N22.3	Up	Tissue	RNA sequencing	308	[46]
RP11-932O9.10	Up				
RP11-572O6.1	Up				
RP11-190C22.8	Up				
RP11-388C12.8	Up				
RP11-160H22.5	Up	Plasma	qRT-PCR	300	[47]
RP11-307C12.11	Up	Tissue	RNA sequencing	308	[48]
RP11-322E11.5	Up	Tissue	RNA sequencing, microarray	687	[49]
RP11-150012.3	Up				
RP11-160H22.5	Up	Plasma	qRT-PCR	567	[50]

Note: FISH, fluorescence *in situ* hybridization; HCC, hepatocellular carcinoma; qRT-PCR, quantitative reverse transcription-polymerase chain reaction.

A case in point is RP11-40C6.2, which stands out as one of the most promising biomarker candidates due to its validation in a clinical cohort study. It is not only consistently elevated in HCC tumors but is also readily detectable in serum—a critical feature for non-invasive liquid biopsies. Clinically, higher serum levels strongly align with aggressive phenotypes, including larger tumor burden, vascular invasion, and poorer survival. This robust clinical association is mechanistically explained by its function in stabilizing the YAP1 oncoprotein within the Hippo pathway, providing a solid rationale for its evaluation in risk stratification and as a potential complement to alpha-fetoprotein (AFP) [28].

In contrast, RP11-286H15.1 behaves as a tumor-suppressive transcript, showing reduced expression in HCC. Its low expression level is associated with inferior survival. Mechanistically, it promotes the ubiquitination and degradation of Poly(A) Binding Protein Cytoplasmic 4 (PABPC4), downregulating oncogenic effectors such as tripartite motif containing 37 (TRIM37) and CDC27. This offers both mechanistic plausibility and biomarker utility. Overexpression studies indicate suppression of proliferation, invasion, and metastasis, suggesting potential for prognostic readouts and therapeutic targeting [29].

Additionally, RP11-466I1.1 has been linked to adverse histopathologic features—including poor differentiation and incomplete encapsulation—which often track with microinvasion and early recurrence risk. This positions the transcript as a prognostic indicator that could refine postoperative surveillance intensity and enrich adjuvant therapy trials. Finally, RP11-284P20.2 enhances c-MET protein synthesis and is associated with proliferative and metastatic phenotypes. Clinically, its higher expression correlates with worse outcomes and may identify MET-driven tumors, suggesting dual roles as a diagnostic adjunct and a putative predictor for MET-targeted strategies [30].

Taken together, these candidates exemplify how RP11 lncRNAs can be developed along a biomarker pipeline—from Formalin-Fixed Paraffin-Embedded (FFPE) and fresh-tissue quantification to minimally invasive assays using quantitative reverse transcription polymerase chain reaction (qRT–PCR) on serum, plasma or extracellular vesicle fractions. Clear next steps include harmonized pre-analytical handling, prespecified cutoffs, independent validation with multivariable models against clinical covariates, and serial sampling pre- and post-resection or during systemic therapy to establish utility in early detection, recurrence surveillance, and treatment selection.

## 5 Metabolic Regulation in HCC

A clear pattern emerging from the study of oncogenic RP11 lncRNAs in HCC is their consistent convergence on the strategic rewiring of glucose metabolism. Rather than targeting disparate pathways, these lncRNAs frequently function to enforce a Warburg-like phenotype. This recurring theme suggests that hijacking core metabolic machinery is a central strategy utilized by this lncRNA family (Table 3).

**Table 3:** RP11-lncRNAs involved in metabolic regulation in HCC

Gene IDs	Metabolic regulation	Platform	References
RP11-817I4.1	miR-3120-3p/ACLY binding (fatty acid synthesis)	Nile red staining, western blot, dual-luciferase reporter assay	[51]
RP11-495P10.1	PDK1/PDH axis (glucose metabolism, NR4A3 repression)	Lactate production assay, qRT-PCR, western blot, acetyl-CoA production assay	[52]
RP11-386G11.10	ZBTB7A/miR-345-3p/HNRNPU (lipid metabolism)	Triglyceride assay, cholesterol assay, nile red staining, lactate assay, qRT-PCR, western blot	[53]
RP11-241J12.3	Pyruvate carboxylase (pyruvate metabolism, DNA repair disruption)	Intracellular metabolites assay, RNA immunoprecipitation, western blot	[54]

Note: qRT-PCR, quantitative reverse transcription-polymerase chain reaction.

**Figure 2:**
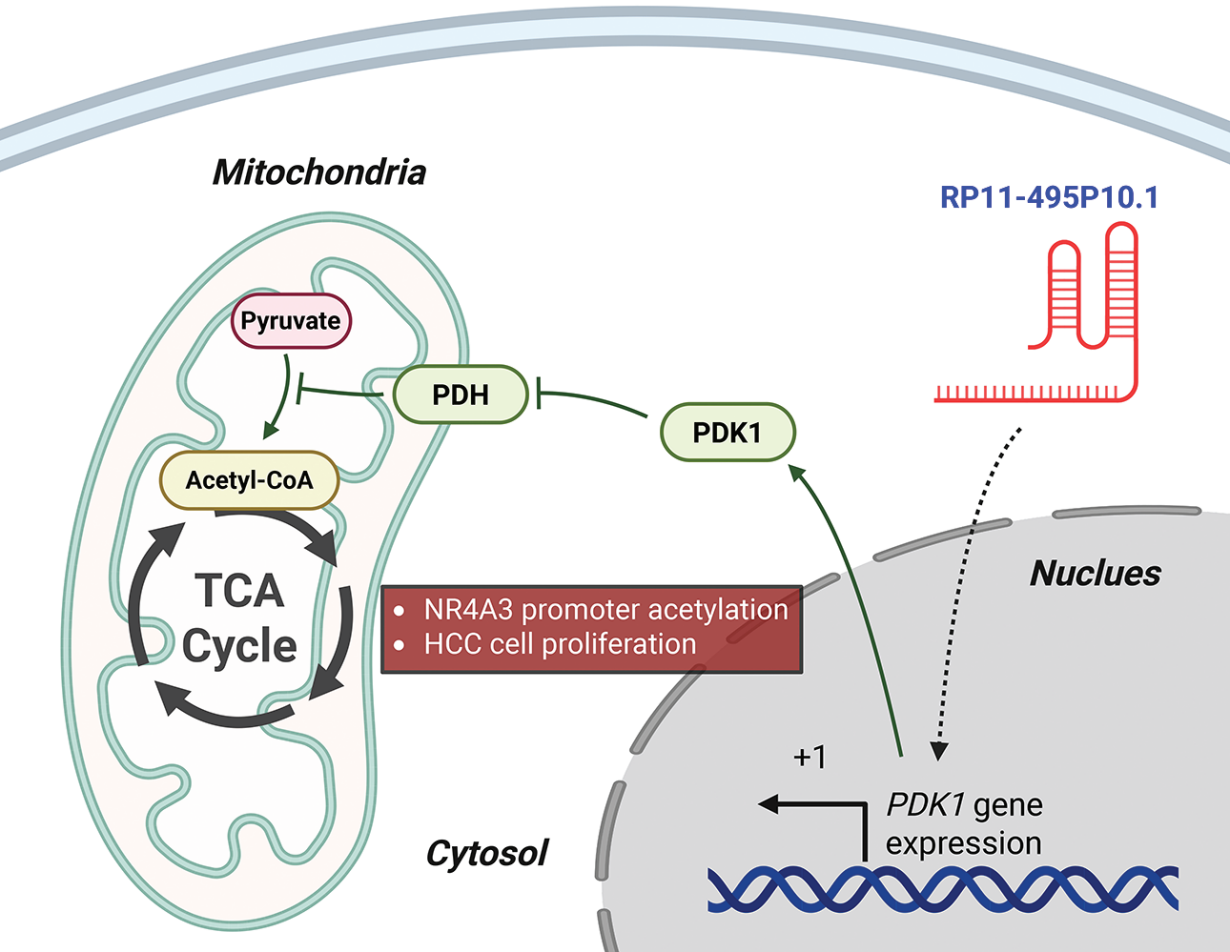
RP11-495P10.1 enforces glycolytic reprogramming via the PDK1–PDH axis in HCC. In the nucleus, RP11-495P10.1 upregulates the transcription of the *PDK1* gene by promoting transcriptional initiation at its transcription start site (+1). The resulting PDK1 protein moves to the mitochondria, where it phosphorylates and inactivates pyruvate dehydrogenase (PDH). This inactivation blocks the conversion of pyruvate to acetyl-CoA, thus reducing carbon entry into the tricarboxylic acid (TCA) cycle. As a result, the cell shifts its metabolism towards glycolysis. This metabolic reprogramming, along with the modulation of NR4A3 promoter acetylation, ultimately drives HCC cell proliferation. Green arrows indicate activation pathways, while blunted red lines indicate inhibition. Created with BioRender.com (Publication license ID: TB28MJEXO8)

For instance, RP11-241J12.3 drives pyruvate carboxylase–dependent anaplerosis, boosting oxaloacetate replenishment to sustain tricarboxylic acid cycle (TCA) cycle throughput and biosynthetic precursor generation while favoring aerobic glycolysis and lactate output. In parallel, its disruption of DNA mismatch repair amplifies replication stress and mutational load, a context that further selects for high-glycolytic, nicotinamide adenine dinucleotide phosphate (NADPH)-buffered states, and its elevated expression associates with larger tumors and advanced stage, consistent with a metabolically hardened phenotype [54]. Similarly, RP11-495P10.1 is a noteworthy metabolic regulator whose functional validation makes it a potential therapeutic target. This lncRNA targets the pyruvate dehydrogenase kinase 1 (PDK1)/pyruvate dehydrogenase (PDH) axis, increasing the inhibitory phosphorylation of PDH to restrict acetyl-CoA entry into mitochondria and divert pyruvate toward lactate. The significance of this oncogenic role is underscored by functional studies where knockdown of RP11-495P10.1 was shown to relieve PDH inhibition and attenuate tumor cell growth, providing strong evidence of its therapeutic potential (Fig. 2) [52].

In addition, RP11-817I4.1 shifts metabolism toward *de novo* lipogenesis by sponging miR-3120-3p to de-repress ACLY. This expands cytosolic acetyl-CoA and citrate–acetyl-CoA cycling for fatty-acid and cholesterol synthesis, which supports membrane biogenesis and motility, thereby promoting rapid proliferation and migration [51]. Finally, RP11-386G11.10 integrates ZBTB7A/miR-345-3p/HNRNPU into a feed-forward circuit that sustains lipogenic gene expression and RNA–protein interactions controlling lipid metabolic transcripts. This stabilizes a high-lipid-flux program that fuels growth and survival under nutrient fluctuation [53]. Collectively, these findings underscore the pivotal and multifaceted roles of RP11 lncRNAs in reprogramming HCC metabolism. They highlight these transcripts not as bystanders but as central regulators that offer promising and specific avenues for therapeutic intervention targeting the unique energetic and biosynthetic dependencies of liver cancer.

## 6 RP11 lncRNAs: miRNA Interactions in HCC

ceRNA regulation refers to RNA–RNA crosstalk in which transcripts that share miRNA response elements compete for the same miRNAs; by sequestering a given miRNA, a lncRNA de-represses the mRNA targets of that miRNA and shifts pathway output [55]. The magnitude and direction of ceRNA effects are context dependent, scaling with relative abundance, binding-site stoichiometry, and subcellular localization [55]. Within this framework, RP11-derived lncRNAs shape oncogenic signaling in HCC primarily through ceRNA-type interactions that titrate miRNAs away from their mRNA targets, thereby derepressing metabolic, cell-cycle, and invasion programs (Table 4).

**Table 4:** RP11-lncRNAs involved in miRNA-mediated regulatory networks in HCC

Gene IDs	miRNA network	Platforms	References
RP11-620J15.3	miRNA (miR-326/GPI, glycolysis)	Dual luciferase reporter assay, RNA immunoprecipitation, qRT-PCR, western blot	[56]
RP11-81H3.2	miRNA (miR-490-3p, Tankyrase 2)	Luciferase reporter assay, qRT-PCR, western blot	[57]
RP11-295G20.2	miRNA (miR-6884-3p/CCNB1)	Luciferase reporter assay, RNA immunoprecipitation assay, qRT-PCR, Spearman’s correlation analysis, western blot	[58]
RP11-20J15.2	E2F2 (ceRNA axis)	Public database-based analysis	[59]
RP11-909N17.2	miRNA (miR-767-3p/SMIM7)	Luciferase reporter assay, qRT-PCR, Spearman’s correlation analysis, western blot	[60]
RP11-422N16.3	miRNA (miR-23b-3p/ZEB1)	Dual-luciferase reporter assay, RNA-pull down assay, qRT-PCR, western blot	[61]
RP11-424C20.2	miRNA (miR-378a-3p/UHRF1)	Public database-based analysis	[62]
RP11-564D11.3	VEGFA (ceRNA axis)	Luciferase reporter assay, RNA immunoprecipitation assay, qRT-PCR, Spearman’s correlation analysis, western blot	[63]
RP11-513I15.6	miRNA (miRNA-1262/ceRNA axis)	Public database-based analysis, qRT-PCR	[64]

Note: ceRNA, competitive endogenous RNA; miRNA, microRNA; qRT-PCR, quantitative reverse transcription-polymerase chain reaction.

**Figure 3:**
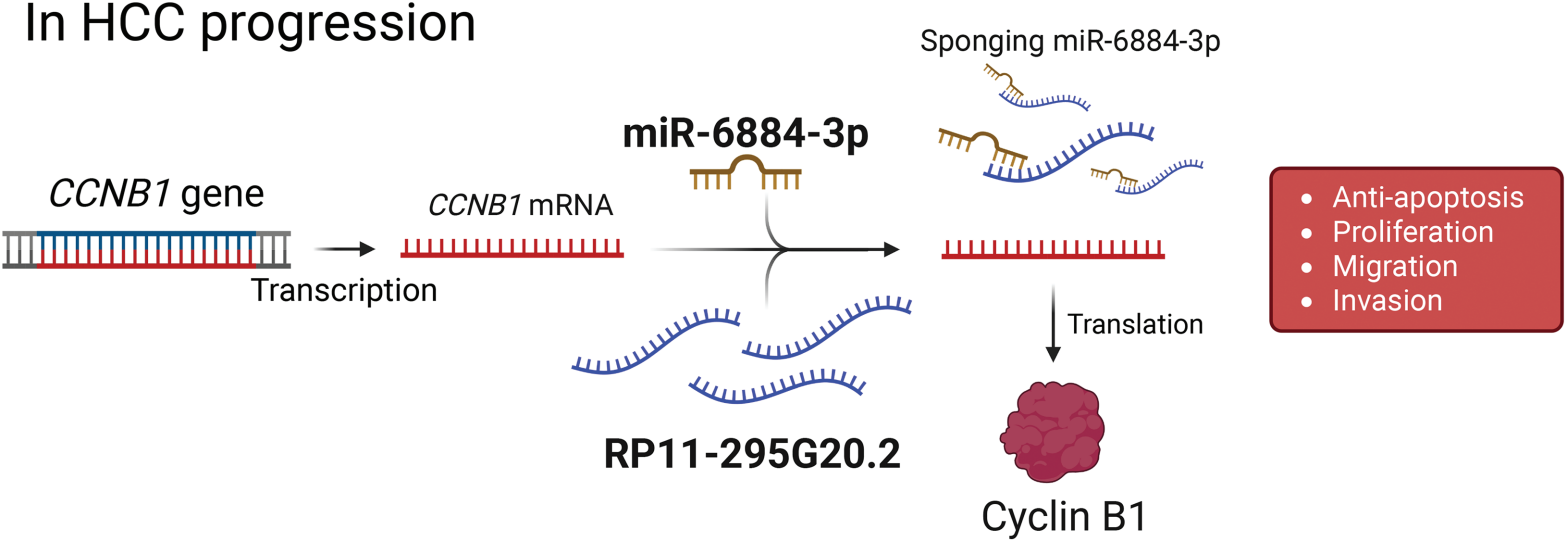
Proposed model of RP11-295G20.2–mediated regulation of CCNB1 in HCC progression. The *CCNB1* gene is transcribed into mRNA, which is a target for suppression by microRNA-6884-3p (miR-6884-3p). The long non-coding RNA RP11-295G20.2 functions as a competing endogenous RNA (ceRNA), or a “sponge”, by binding to and sequestering miR-6884-3p. This action prevents the miRNA from binding to *CCNB1* mRNA, leading to increased translation of the Cyclin B1 protein. Elevated levels of Cyclin B1 drive key tumorigenic processes in hepatocellular carcinoma (HCC), such as proliferation, migration, invasion, and inhibition of apoptosis. Created with BioRender.com (Publication license ID: BC28MJTYIW)

For example, RP11-295G20.2 engages the miR-6884-3p/CCNB1 axis, where miR-6884-3p normally constrains Cyclin B1; by acting as a sponge, RP11-295G20.2 lifts this brake to boost CCNB1, facilitating G2/M transition through CDK1 activation and thereby accelerating cell-cycle progression (Fig. 3) [58]. In addition to directly regulating the cell cycle, other RP11 lncRNAs utilize the same ceRNA strategy to rewire cancer metabolism. For instance, RP11-620J15.3 is markedly upregulated and sequesters miR-326 to elevate glucose-6-phosphate isomerase (GPI), an enzyme that converts glucose-6-phosphate to fructose-6-phosphate near the top of glycolysis [56].

Moreover, RP11-81H3.2 targets miR-490-3p, which physiologically restrains tankyrase 2 (TNKS2), a positive regulator of Wnt/β-catenin signaling via AXIN degradation. By binding miR-490-3p, RP11-81H3.2 increases TNKS2 abundance, potentiates β-catenin–dependent transcription, and drives proliferation, migration, and invasion consistent with epithelial–mesenchymal transition (EMT)-like phenotypes [57].

These findings highlight the critical roles of RP11 lncRNAs in miRNA-mediated regulatory networks. By sponging specific miRNAs, these lncRNAs contribute to HCC pathogenesis and represent potential targets for RNA-based therapeutic interventions.

## 7 Therapeutic Targeting of RP11-Derived lncRNAs in HCC

The central role of RP11-derived lncRNAs in driving HCC pathogenesis makes them attractive therapeutic targets. Their frequent tumor-specific expression and critical functions in maintaining malignant phenotypes offer a potential therapeutic window with fewer side effects than conventional chemotherapy [65]. Current and emerging strategies to modulate lncRNAs can be categorized into oligonucleotide-based approaches, genome editing tools, and small molecule inhibitors.

**Figure 4:**
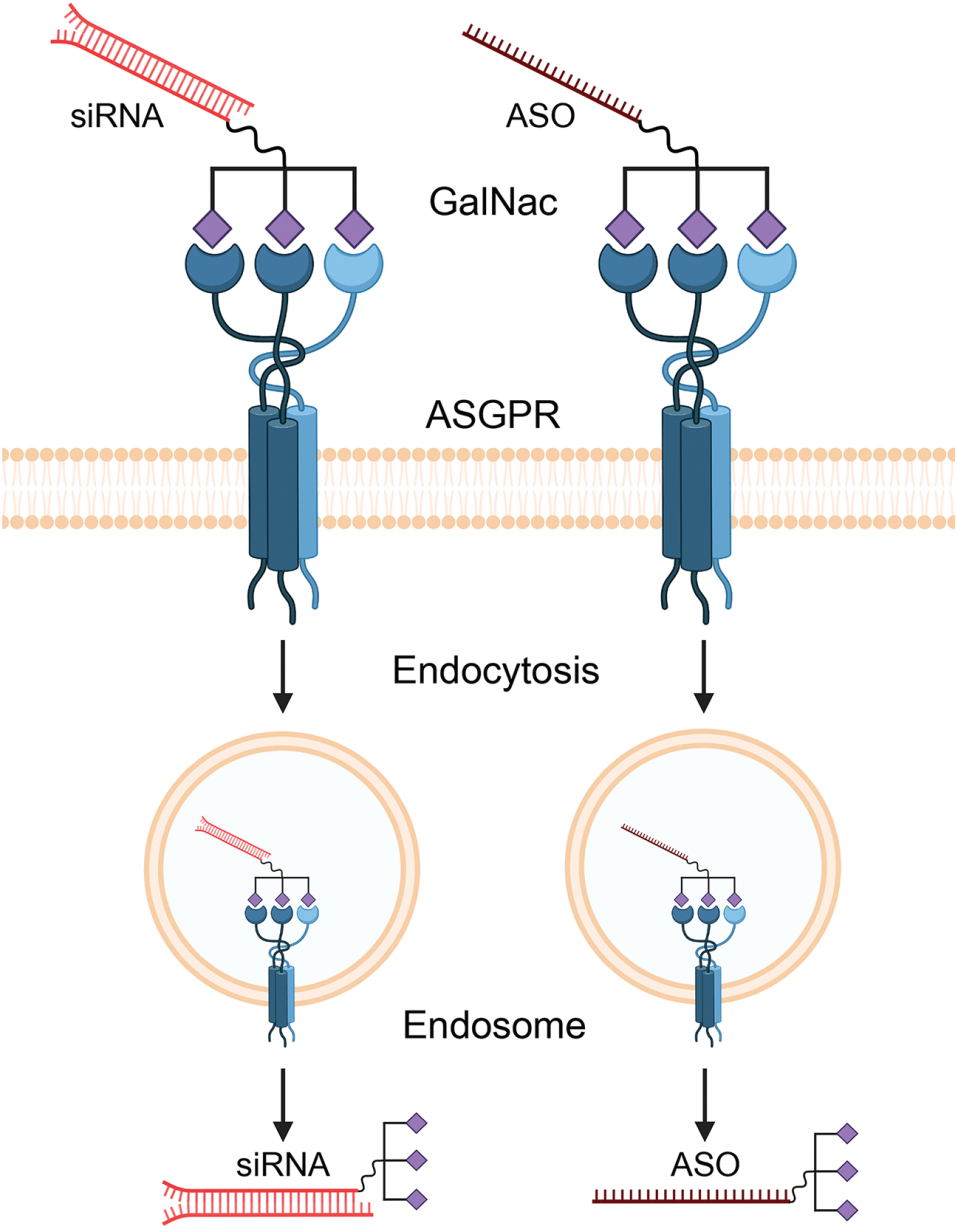
Mechanism of GalNAc-mediated delivery of therapeutic oligonucleotides to hepatocytes. Therapeutic agents like small interfering RNAs (siRNAs) and antisense oligonucleotides (ASOs) are conjugated with N-acetylgalactosamine (GalNAc) ligands. This GalNAc-conjugate specifically binds to the asialoglycoprotein receptor (ASGPR), which is abundantly expressed on the surface of liver cells (hepatocytes). This high-affinity binding triggers receptor-mediated endocytosis, causing the cell to internalize the entire therapeutic complex into an endosome. Inside the cell, the siRNA or ASO is released from the endosome into the cytoplasm, where it can find and degrade its target lncRNA. Created with BioRender.com (Publication license ID: SP28N9FM2V)

Antisense oligonucleotides (ASOs) and small interfering RNAs (siRNAs) represent the most advanced strategies for targeting lncRNAs [66]. ASOs are single-stranded synthetic nucleic acids that bind to a target lncRNA via Watson-Crick base pairing, leading to its degradation by RNase H [67]. siRNAs are double-stranded molecules that engage the RNA-induced silencing complex (RISC) to cleave the target lncRNA (Fig. 4) [68].

For oncogenic RP11 lncRNAs that are upregulated in HCC—such as RP11-495P10.1, which promotes glycolysis via the PDK1/PDH axis [52], or RP11-284P20.2, which enhances c-MET synthesis [32]—targeted ASOs or siRNAs could effectively deplete the transcript and reverse its pro-tumorigenic effects. Although clinical studies directly targeting RP11 lncRNAs have not yet been initiated, preclinical studies targeting other lncRNAs in HCC (e.g., MALAT1) have shown promising results in suppressing tumor growth and metastasis, providing proof-of-concept for this approach.

A primary challenge is the efficient and tumor-specific delivery of these oligonucleotides *in vivo*. For liver-targeted therapies, N-acetylgalactosamine (GalNAc) conjugation has emerged as a groundbreaking solution [69]. GalNAc is a ligand with high affinity for the asialoglycoprotein receptor (ASGPR), which is abundantly and almost exclusively expressed on the surface of hepatocytes [55]. Conjugating GalNAc to ASOs or siRNAs enables selective targeting to the liver and efficient uptake by liver cells after subcutaneous injection. This technology underpins several FDA-approved RNA therapeutics, such as Patisiran, Givosiran, and Inclisiran, establishing its clinical validity [70]. The GalNAc delivery platform is therefore a highly promising strategy for developing therapeutics against RP11 lncRNAs in HCC.

For more durable, locus-level repression, the CRISPR-Cas9 system can be adapted to repress gene expression without inducing DNA breaks [71]. Using catalytically inactive Cas9 (dCas9) fused to a transcriptional repressor domain like KRAB, and guided by a specific RNA to the promoter of an oncogenic RP11 lncRNA [72], the CRISPR interference (CRISPRi) approach can epigenetically silence the target gene. This strategy offers potent and long-lasting inhibition, making it particularly suitable for silencing the transcription of key oncogenic lncRNAs in HCC cells.

Despite this promise, significant hurdles remain for lncRNA-based therapeutics, including delivery, stability, and off-target effects. However, rapid advancements in nanoparticle-based delivery systems and chemical modifications of oligonucleotides are paving the way for clinical translation.

## 8 Conclusion

This review has critically synthesized the evidence to establish that the roles of RP11-derived lncRNAs—so named for their clone-based origins—are not a random collection of functions but rather converge on core oncogenic strategies [2]. Drawing from the standard lncRNA toolkit of molecular mechanisms used across cancers, we have shown that their impact in HCC clusters along key translational axes, most notably the strategic rewiring of cancer metabolism and the deployment of ceRNA networks to dismantle tumor-suppressive miRNA pathways [1,26,27]. By moving beyond a simple catalog and identifying these convergent mechanisms, this review provides a new conceptual framework for understanding RP11 lncRNAs as functionally clustered, rather than disparate, regulators in hepatocarcinogenesis.

Despite this emerging clarity, significant gaps and challenges remain before these lncRNAs can be fully translated into clinical practice. Many RP11 loci are still known only by their legacy symbols; isoform complexity, context dependence, and subcellular localization continue to confound definitive mechanism-of-action claims. A salient example of this complexity is RP11-363E7.4, which acts as a tumor suppressor in gastric cancer but is paradoxically associated with poor prognosis in bladder cancer, suggesting a functional switch depending on the cancer type. Its role in HCC is further nuanced, with its expression being dynamically altered by therapeutic stress. This functional plasticity underscores that a “one-size-fits-all” label is insufficient for many RP11 lncRNAs. Furthermore, assay variability limits cross-study comparability. Progress will hinge on isoform-aware single-cell and spatial maps to fix cell-type context, quantitatively validated models of ceRNA stoichiometry, and standardized prospective pipelines—harmonizing pre-analytics, prespecified cutoffs, and multivariable benchmarks—with serial sampling across surgery and therapy [73]. As we have discussed, developing targeted therapies against these critical lncRNAs, ranging from clinically advanced oligonucleotide drugs to emerging genome editing tools, represents a vital and promising frontier for future HCC treatment.

Most importantly, the RP11 catalog remains only partially mined: among thousands of transcripts, a substantial fraction are likely “hidden treasures” with unappreciated roles in metabolism, immune evasion, and treatment response. Furthermore, while our review focused on the functions of individual lncRNAs, future studies should investigate the potential for crosstalk between different RP11 lncRNA networks, which may form a higher-order regulatory web that collectively orchestrates HCC progression. As isoform-aware analytics and clinically deployable RNA assays mature, these understudied RP11 lncRNAs are poised to become hot topics—both as mechanistic entry points into HCC biology and as next-generation biomarkers and therapeutic targets capable of refining detection, guiding therapy selection, and improving outcomes.

## Data Availability

Not applicable.

## Abbreviations

**Abbreviation**: **Full Name**
ACLY: ATP Citrate Lyase
AFP: Alpha-Fetoprotein
ASGPR: Asialoglycoprotein Receptor
ASO: Antisense Oligonucleotide
BAC: Bacterial Artificial Chromosome
CCNB1: Cyclin B1
CDC27: Cell Division Cycle 27
CDK1: Cyclin-Dependent Kinase 1
ceRNA: Competing Endogenous RNA
CoA: Coenzyme A
CRISPRi: CRISPR Interference
dCas9: Catalytically Inactive Cas9
EMT: Epithelial–Mesenchymal Transition
FFPE: Formalin-Fixed Paraffin-Embedded
FISH: Fluorescence *In Situ* Hybridization
GalNAc: N-Acetylgalactosamine
GPI: Glucose-6-Phosphate Isomerase
HCC: Hepatocellular Carcinoma
HIF1A: Hypoxia-Inducible Factor 1 Alpha
HNRNPU: Heterogeneous Nuclear Ribonucleoprotein U
KRAB: Krüppel-Associated Box
lncRNA: Long Non-Coding RNA
MET: MET Proto-Oncogene, Receptor Tyrosine Kinase
miRNA: MicroRNA
mRNA: Messenger RNA
NADPH: Nicotinamide Adenine Dinucleotide Phosphate (Reduced Form)
NR4A3: Nuclear Receptor Subfamily 4 Group A Member 3
PABPC4: Poly(A) Binding Protein Cytoplasmic 4
PDH: Pyruvate Dehydrogenase
PDK1: Pyruvate Dehydrogenase Kinase 1
qRT–PCR: Quantitative Reverse Transcription Polymerase Chain Reaction
RISC: RNA-Induced Silencing Complex
RNase H: Ribonuclease H
RPCI: Roswell Park Cancer Institute
RP11: Roswell Park Cancer Institute Human BAC Library 11
siRNA: Small Interfering RNA
TCA: Tricarboxylic Acid Cycle
TNKS2: Tankyrase 2
TRIM37: Tripartite Motif Containing 37
VEGFA: Vascular Endothelial Growth Factor A
ZEB1: Zinc Finger E-Box Binding Homeobox 1
ZBTB7A: Zinc Finger and BTB Domain Containing 7A
